# CircRNA-CIRH1A Promotes the Development of Osteosarcoma by Regulating PI3K/AKT and JAK2/STAT3 Signaling Pathways

**DOI:** 10.1007/s12033-023-00812-0

**Published:** 2023-08-23

**Authors:** Meng Zhang, Xiang Wang, Jianfeng Zhao, Jizhou Yan, Xiaodan He, Danxia Qin, Fang Liang, Kai Tong, Jianjian Wang

**Affiliations:** 1 Department of Thoracic and Bone-Soft Tissue Surgery, Hubei Cancer Hospital, Tongji Medical College, HuaZhong University of Science and Technology, Wuhan, 430079 China; 2 Department of Orthopedics, Fuyang Traditional Chinese Medicine Orthopedics Hospital, Hangzhou, 311400 Zhejiang China; 3 Department of Orthopedics, Beijing University of Chinese Medicine Affiliated Dongzhimen Hospital, Beijing, 100700 China; 4 Department of Orthopedics, Renmin Hospital of Wuhan University, Wuhan, 430060 China

**Keywords:** circRNA-CIRH1A, Osteosarcoma, PI3K, AKT, JAK2, STAT3

## Abstract

Osteogenic sarcoma (OS), one of the mesenchymal tumors with a high degree of malignancy, mainly occurs in the metaphysis of the long bones and around the knee joints in children and adolescents. The poor diagnosis in patients with OS can be attributed to the lack of early clinical symptoms, although the growth of tumor mass gradually results in severe pain and systemic symptoms. The mechanisms underlying the pathogenesis of OS are not fully understood. Thus, identifying early diagnostic biomarkers and novel targets involved in the progression of OS is of critical significance in the management of OS. CircRNA is a class of non-coding RNAs characterized by the close-loop structure and increased stability, which are implicated in the regulation of cell proliferation, differentiation, migration, and apoptosis. Moreover, circRNAs also play significant roles in aging and chronic disorders, such as cancer and cardiovascular diseases. Accordingly, we reported the upregulation of circRNA-CIRH1A in OS tissues and cell lines. Silencing circRNA-CIRH1A in OS cell lines (U2OS, HOS, Saos-2, and MG-63) could inhibit the cell proliferation, invasion, migration, and apoptosis, which was also validated in xenograft tumorigenesis mouse model. We further demonstrated that circRNA-CIRH1A sponged miR-1276, which subsequently disrupted the effect of miR-1276 on PI3K/AKT and JAK2/STAT3 signaling pathways. Together, our study revealed the oncogenic role of circRNA-CIRH1A in OS, and identified miR-1276/ PI3K-AKT and JAK2-STAT3 signaling axis as the key downstream mediators of circRNA-CIRH1A.

**Supplementary Information:**

The online version contains supplementary material available at 10.1007/s12033-023-00812-0.

## Introduction

Osteosarcoma (OS) is a primary malignant tumor originated in the skeleton, which is characterized by the production of osteoid tissues or immature bone tissues [1]. In general, OS is frequently initiated from mesenchymal tissues and develops in the epiphysis of the long diaphysis (distal femur and proximal tibia) of adolescents, with a male-to-female ratio of about 2:1. Notably, the early symptoms of OS are not apparent, primarily manifested as intermittent dull pain [2, 3]. However, the progression of tumor mass gradually results in severe pain and systemic symptoms, which ensue with distant tissue metastases and the pathological fracture. Although efforts have been made in exploring the mechanisms of OS progression, the exact etiology and contributing factors of OS are currently unclear. Previous studies indicated that OS was characterized by malignant spindle stromal cells and large amounts of sarcomatoid stroma, which comprise of chondrocytes and poorly differentiated tumor cells [4–6]. The five-year survival rate of OS patients is less than 20% with conventional surgery, and chemotherapy could extend the overall survival rate of patients with OS [5, 6]. However, the development of drug resistance and cancer recurrence limits the treatment outcome of chemotherapy. It is of great clinical significance to identify novel therapeutic targets for novel drug development and biomarkers for early diagnosis [7, 8].

In recent years, the application of high throughput sequencing technologies has identified the deregulation of different classes of non-coding RNAs in pathological conditions. CircRNA has gained increasing attention due to its stable close-loop structure and widely reported roles in regulating gene expression [9]. CircRNAs are by-products of mRNA splicing and are mainly distributed in the cytoplasm of eukaryotic cells [10]. Previous studies indicated that circRNAs can function as competing endogenous RNAs (ceRNAs) to sponges microRNAs and regulate the expression of downstream mRNAs [11–13]. Notably, circRNAs are implicated in the regulation of proliferation, invasion, and apoptosis of various tumor cells [10–15].

Here, we reported that circRNA-CIRH1A was upregulated in osteosarcoma tissues and cell lines. In the study of 40 pairs of OS tissues and para-cancerous normal tissues, we found that circRNA-CIRH1A (circ_0007018) expression was significantly higher in OS tissues. Silencing circRNA-CIRH1A suppressed cell proliferation, invasion and migration, and promoted apoptosis. We further demonstrated that circRNA-CIRH1A sponged miR-1276, which subsequently disrupted the effect of miR-1276 on PI3K/AKT and JAK2/STAT3 signaling pathways. PI3K/AKT signaling pathway plays a significant role in regulating the proliferation and survival of various tumor cells, such as liver cancer, breast cancer, and osteosarcoma [16]. Moreover, this signaling cascade is closely related to the invasion and metastasis of tumor cells, as well as the angiogenesis in tumor tissues [17, 18]. PI3K/AKT regulates cellular functions through a variety of downstream effectors, such as the activation of JAK2/STAT3 signaling pathway by phosphorylating STAT3. JAK2/STAT3 belongs to non-receptor tyrosine kinase, which plays a vital role in the proliferation and apoptosis of cancer cells [19–22]. In our study, we revealed the oncogenic role of circRNA-CIRH1A in OS by targeting miR-1276 and regulating PI3K/AKT and JAK2/STAT3 signaling pathways.

## Materials and Methods

### Tissue Collection

The study was approved by the Medical Ethics Committee at the Fuyang Traditional Chinese Medicine Orthopedics Hospital (2019-F027). OS tumor tissues and adjacent normal tissues were collected from 40 patients diagnosed with OS at the Fuyang Traditional Chinese Medicine Orthopedics Hospital from June 2018 to Dec 2019. Patient samples were obtained after written informed consent had been obtained. Specimens were immediately frozen in liquid nitrogen after resection and stored at − 80 °C until usage.

### Cell Culture, Transfection and Stable Knockdown Cell Generation

Human osteoblast cell lines, such as U2OS, HOS, Saos-2, and MG-63, were purchased from Hengya Biotechnology (Shanghai, China). All the cell lines were cultured in DMEM high glucose medium containing 10% FBS (Hyclone, CA, USA), and 1% penicillin/streptomycin (Hyclone, CA, USA) under the condition of 37 °C and 5% CO2. Cells were harvested for experiments in exponential growth state. Cell transfection was performed using Lipofectamine® 3000 reagent (Invitrogen, CA, USA). In 6-well plates, 60% confluent cells were transfected with 100 nM of microRNA mimic or inhibitor or siRNA based on manufacturer’s instruction. Transfected cells were subjected to subsequent analysis 48 h post-transfection.

The lentivirus carrying shRNAs targeting circRNA-CIRH1A were synthesized by Genscript Biotech company (Nanjing, China). Cells were transduced with lentivirus carrying control shRNA or shRNA targeting circRNA-CIRH1A at a MOI (multiplicity of infection) = 5 in the presence of 10 µg/ml polybrene (Sigma, Germany). Infected cells were selected with 1.0 μg/mL puromycin (Sigma, Germany) for two weeks to eliminate the uninfected cells before further experiment.

### Cell Counting Kit (CCK)-8 ASSAY

Cells were transfected with siRNA targeting circRNA-CIRH1A or its control. 48 h after transfection, cells were seeded in to a 96 -well plate at a density of 1500 cell/well and cultured in a humidified cell culture incubator for 0, 24, 48, and 72 h, respectively. Subsequently, 10 μL CCK8 reaction solutions (Beyotime, Shanghai, China) was added to the cell culture at indicated time point and incubated for 1 h in a humidified cell culture incubator. The light absorption value (OD value) in each condition was captured at 450 nm wavelength on a Synergy H1 microplate reader.

### RNA Extraction and Real-Time PCR

200 mg of tissue or 1 million of cells were used from RNA extraction in 1 mL of Trizol (Beyotime, Shanghai, China) for 5 min. Further, 0.2 mL of chloroform was added in to the samples and the samples were shaken gently for 30 s and incubated for 5 min at room temperature. After centrifugation for 15 min, and the layer of RNA samples were isolated. An equal volume of isopropanol was added to precipitate the RNA and the mixture was centrifuged again for 15 min and the precipitated RNA samples were dissolved in RNase-free water.

1 μg of total RNA was used for reverse-transcription into cDNA using RevertAid First Strand cDNA Synthesis Kit (Thermo Fisher Scientific, CA, USA). The resulted cDNA was diluted to 40 ng/μL and analyzed in a 7500 Real-Time PCR System (Applied Biosystems, CA, USA) using SYBR premix EX TAQ II kit (Takara, Dalian, China). The PCR cycling condition used: 95 °C for 5 min, then 40 cycles of 95 °C for 10 s and 60 °C for 30 s, followed by a dissolution curve of 95 °C for 15 s, 60 °C for 30 s, and 95 °C for 15 s. All the primers were designed using Primer Blast function of NCBI, and GAPDH was used as the internal reference for gene expression normalization. The related primers are as follows:

circRNA-CIRH1A: 5′-TGAGTCTCGGGCTACAGAAG-3′ (forward) and 5′-GCA TACTTGATGTTTAACGCCTG-3′ (reverse).

GAPDH: 5′-GGAGCGAGATCCCTCCAAAAT-3′ (forward) and 5′-GGCTGTTGTCATACTTCT CATGG-3′ (reverse).

U6: 5’-GCTTCGGCAGCACATATACTAAAAT-3′ (forward) and 5′-CGCTTCACGAATTTGCGTGTCAT-3’ (reverse).

### Protein Extraction and Western Blotting

Total protein from tissues and cells (approximately 1 million cells) were collected using RIPA lysis buffer containing protease inhibitor cocktail (Thermo Fisher Scientific, CA, USA). Cells suspended in RIPA buffer were lysed on ice for 10 min and the lysates were centrifuged at 14,000 rpm for 10 min. The supernatant was quantified by a BCA Protein assay kit (Beyotime Biotechnology, Shanghai, China). Protein sample was mixed with 5% SDS-PAGE loading buffer and heated at 100℃ for 5 min. 10 µg protein was used for SDS-PAGE electrophoresis and separated protein in SDS-PAGE gel was transferred onto the PVDF membrane (BioRad, CA, USA). After blocking with 5% skimmed milk for 1 h, the membrane was incubated with primary antibodies overnight at 4℃: GAPDH (#2118S), AKT (#4691), phospho-AKT (#4060), PI3K (#4255) (1:1000, Cell Signaling Technologies MA, USA), and phospho-PI3K antibody (1:1000, AF3241, Affinity Biosciences, OH, USA). The membrane was washed 3 times with TBST and further incubated with HRP-linked secondary antibody (1:3000, #7074, Cell signaling Technologies) for 1 h. The protein bands were visualized using an enhanced chemiluminescence kit (Santa Cruz, TX, USA) and photographed on a gel imager system (Bio-Rad, CA, United States). The GAPDH was used as the loading control for Western blot quantification.

### 5-Ethynyl-2′-Deoxyuridine (EDU) Incorporation Assay

Cells (2 × 10^5^ per well) were inoculated in the 24-well plates, and EDU staining kit (Click-iT® EdU, Invitrogen) was used for Edu incorporation assay. When cells reached 80%, the culture medium was replaced with the medium containing 1× EdU and incubated for 2 h. After rinsing by PBS, 4% formaldehyde was added to fix cells for a 15 min, followed by 20 min incubation with 0.5% Triton X-100 in PBS. Then, Click-iT reaction mixture (0.5 mL) added to the fixed cells for 30-min incubation. The stained cells were washed by PBS with 3% BSA, and counterstained with DAPI (1 g/mL) for 15 min in the dark environment. After staining and wash with PBS, the images were captured under Leica AM6000 microscope.

### Flow Cytometry Analysis

Cells were trypsinized and washed twice with PBS, and re-suspended in the Annexin-V binding buffer. The detection of cell apoptosis was performed using the FITC Annexin-V Apoptosis Detection Kit (BD Biosciences, PharMingen, San Jose, CA, USA) according to the manufacturer's instructions. In brief, 5 μL Annexin V-FITC and 5 μL PI were added to the 1000 μL cell suspension with 1 million cells and incubated for 30 min in the dark. Stained cells were centrifuged and washed twice with Annexin-V binding buffer and resuspended in 400μL Annexin-V binding buffer. The percentage of apoptotic cells was detected by BD FACS CantoTM II Flow Cytometer (BD Biosciences).

### RNase R Digestion

RNase R (TaKaRa, Maebashi, Japan) was used to treat the RNA samples. The RNA sample from 1 million cells was divided equally into two portions: one was used for Rnase R digestion (Rnase R group, 10 unit of RNase R), and the other was used as control (Mock group). The two portions of samples were incubated at 37℃ for 25 min. The relative amount of CIRH1A mRNA and circRNA-CIRH1A in each sample was detected by RT-qPCR.

### RNA Pull-Down Experiment

Cell lysates from approximately 1 million of U2OS and MG63 cells were collected by IP lysis buffer (Beyotime, Shanghai, China) and were incubated biotinylated circRNA-CIRH1A oligo and Control oligos. 10% of the lysates was saved as the input. The mixture was further incubated with M-280 streptavidin magnetic beads (Sigma-Aldrich, Germany) at 4℃ shaking overnight. A magnetic bar was used to pull-down the magnetic beads and associated nucleic acids. Both the input and elutes from the pull-down were purified with Trizol reagent (Invitrogen) according to the manufacturer's protocol. The relative levels of candidate miroRNAs were detected in the pull-down sample, which was normalized to their levels in the input samples.

### Migration and Invasion Experiments

Cell migration as assessed by wound-healing assay. Cells (about 0.2 million) were seeded in 6-well plates for about 80% confluence. A scratch wound was created using a sterile 200 μL pipette tip in the central region of each well. The cells were incubated at 37 °C for 24 h. Cell images were captured using an inverted light microscope (Leica AM6000 microscope). The migration distance was analyzed is using Image J software. The migration rate is calculated as ratio of would distance at 24 h / would distance at 0 h.

Cell invasion was assessed by Transwell invasion assay. The transwell upper chamber (Corning, NY, USA) was coated with Matrigel (BD Biosciences, Bedford, MA, USA), and 5 × 10^5^ cells were inoculated into the transwell upper chamber in serum-free medium. 500 μL of 10% serum-containing medium was added to the lower chamber. After 18 h, culture medium was discarded and the cells were fixed with 4% paraformaldehyde at room temperature for 10 min and stained with 0.5% crystal violet (Sigma, Germany) for 20 min. Cells were photographed under Leica AM6000 microscope.

### Dual-Luciferase Reporter Assay

To demonstrate the functional interaction between circRNA-CIRH1A and miR-1276, the sequence containing the wild type binding site and the sequence with mutated binding site were cloned into the PmirGLO vector. The reporter plasmid and Renilla luciferase (hRlucneo) control plasmid were co-transfected into 5 × 10^5^ cells seeded in 24-well plates, in the presence of either miR-1276 mimic or miR-NC using Lipofectamine 3000 reagent (Invitrogen). 48 h post-transfection, the relative luciferase activities were measured using Dual-Luciferase Reporter Assay Kit (Promega) on a luminescence microplate reader.

### Mice Subcutaneous Tumor Model

All animal procedures were approved by the animal care and use ethical committee of Fuyang Traditional Chinese Medicine Orthopedics Hospital. Twelve male immunodeficient nude mice (30–40 g) were randomly divided into two groups (6 mice in each group): (1) NC group (injected with U2OS cells transfected with si-NC), (2) si-circRNA-CIRH1A (injected with U2OS cells transfected with si-circRNA-CIRH1A). 0.2 mL of cell suspension containing 1 × 10^7^ cells was injected into the flank of each mice. Tumor volume was monitored for 40 days post-injection. On day 40 after tumor cell inoculation, all the mice were euthanized by CO2 asphyxiation. Death was assured by subsequent cervical dislocation and the xenograft tumor of terminally dead mice were resected for weight measurement.

### Immunohistochemical Staining

The tumor tissues were dehydrated, paraffin-embedded, and cut into 5 µm thick slices. Further, the tissue sections were treated with pepsin for 10 min at room temperature. After washing with PBS, the endogenous peroxidase was inactivated with 3% H_2_O_2_ water for 30 min. The tissue sections were blocked by adding 150 µL of goat serum solution at room temperature for 1 h, and incubating the primary antibody at 4 °C overnight. Then, 2–4 drops of HRP-conjugated secondary antibody were added to the tissues for 1 h incubation, which was followed by the addition of 400 µL SignalStain® substrate (Cell Signaling Technologies). In the end, the cell nuclei were counterstained with hematoxylin for 5 min and the section was washed with double-distilled water for 15 min. Section was mounted with cover slips using the mounting medium (Cell Signaling Technologies) before imaging.

### Statistical Analysis

All data were expressed as mean ± standard deviation (SD) and analyzed by SPSS19.0 and Prism9 software. The association between expression level of circRNA-CIRH1A and the clinic pathological parameters was evaluated with Chi-square analysis. The statistical difference between two groups was compared using unpaired student’s t tests. Comparisons among multiple groups were analyzed using one-way analysis of variance (ANOVA) with Tukey’s post hoc test for pairwise comparison. Comparisons of data at multiple time points were examined using two-way ANOVA. Kaplan Meier Curve and log-rank test were used to compare the cumulative survival rates in 90 BC patients. Data were reported as the mean ± standard deviation (SD). *p* < 0.05 was considered to be statistically different.

## Results

### CircRNA-CIRH1A (circ_0007018) is Upregulated in OS Tissues and Cell Lines

**Fig. 1:**
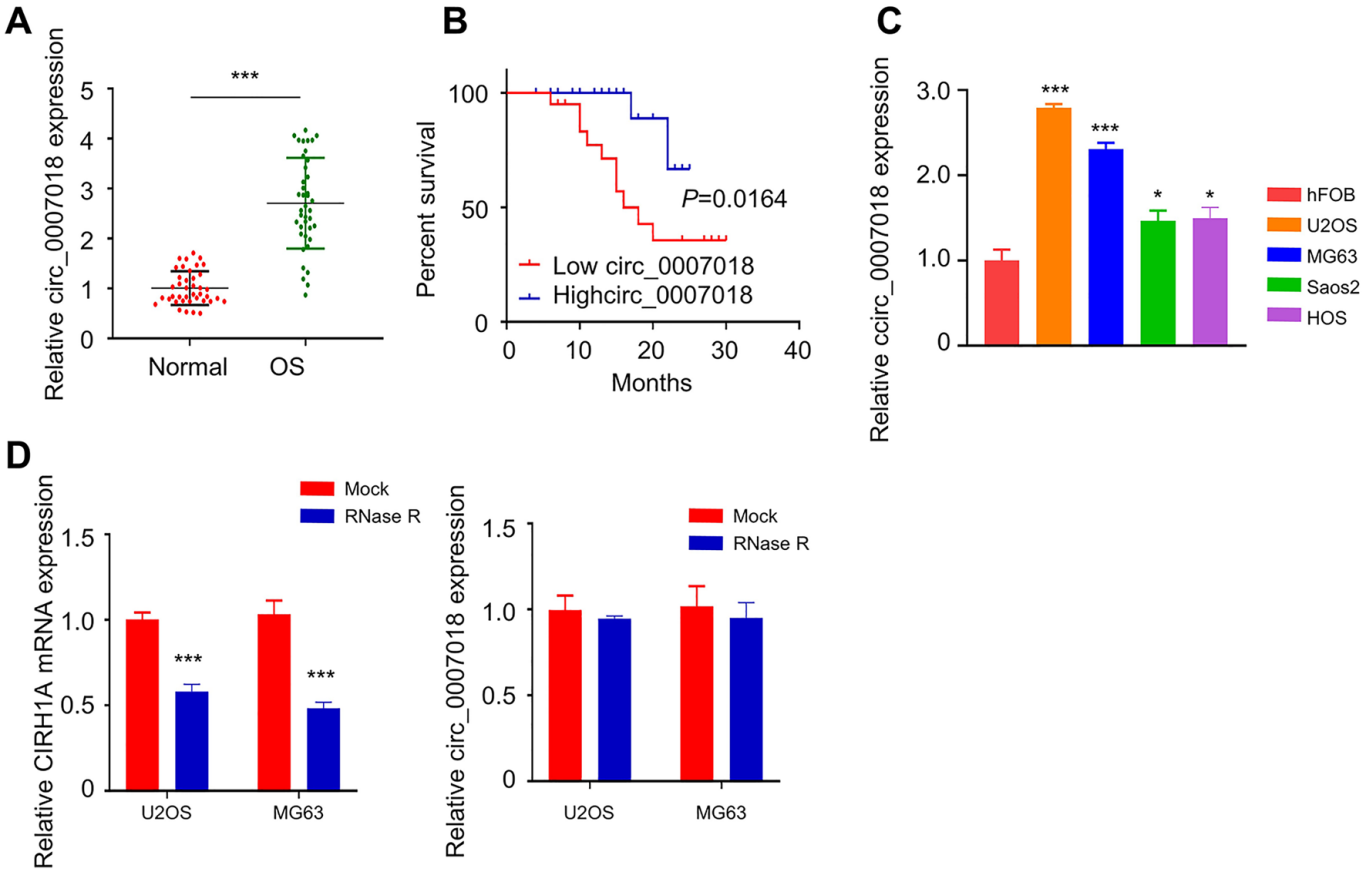
CircRNA-CIRH1A (circ-0007018) was upregulated in OS cancer tissues and cell lines. **A** Circ-CIRH1A expression level was significantly increased in tumor samples from 40 OS patients. **B** Kaplan–Meier survival curve analysis of the overall survival of circRNA-CIRH1A-high and CircRNA-CIRH1A-low-expression patients. **C** The relative expression levels of circRNA-CIRH1A in OS cell lines (U2OS, HOS, Saos-2, and MG-63) and osteoblast cell line (hFOB) was measured by qRT-PCR. **D** The relative CIRH1a-mRNA and circRNA-CIRH1A expressions after RNase R treatment was measured by qRT-PCR. *p < 0.05, **p < 0.01, ***p < 0.001

The expression level of circRNA-CIRH1A (circ_0007018) was detected in 40 pairs of OS cancer tissues and adjacent normal tissues by qRT-PCR (Fig. 1A, *p* < 0.001). It was observed that the expression level of circRNA-CIRH1A was significantly higher in OS tissues as compared to that in adjacent tissues. Based on the median expression value of circRNA-CIRH1A in OS tissue, 40 OS patients were divided into low-expression and high-expression groups (*n* = 20 in each group). Table 1 summarized the associations between expression level of circRNA-CIRH1A and the clinic pathological parameters, including age, gender, tumor sizes and clinical stages, chemotherapeutic regimen, and initial metastasis. A high circRNA-CIRH1A expression was significantly associated with lager tumor size and more advanced clinical stages. The Kaplan–Meier survival curve was employed to evaluate the overall survival rate of two groups (Fig. 1B, *p* = 0.016). The analysis showed that high-expression of circRNA-CIRH1A was associated with a poorer prognosis in OS patients. We also analyzed the relative expression level of circRNA-CIRH1A between OS cell lines (U2OS, HOS, Saos-2, and MG-63 OS) and human osteoblast line by qRT-PCR (Fig. 1C, *p* < 0.001 or *p* < 0.05). The level of circRNA-CIRH1A was significantly higher in all OS cell lines. To validate the close-loop structure of circRNA-CIRH1A, the relative level of cirH1A mRNA and circRNA-CIRH1A in U2OS and MG-63 cells were examined after RNase R digestion by qRT-PCR. The result showed that mRNA level of CIRH1A was decreased after RNase R treatment (*p* < 0.001), while the level of circRNA-CIRH1A remained unchanged (Fig. 1D).

**Table 1:** Correlations of circ_0007018 expressions with clinicopathologic features of osteosarcoma

Factors	circ_0007018 expression		*p* value
	Low (n = 20)	High (n = 20)	
Age		0.744	
≤ 20	12	13	
> 20	8	7	
Sex			0.1967
Male	14	10	
Female	6	10	
Tumor size		0.0285	
≤ 5 cm	8	2	
> 5 cm	12	18	
Tumor location		0.2881	
Tibia/femur	16	13	
Elsewhere	4	7	
Clinical stage		0.0326	
I	11	3	
II	6	6	
III	1	5	
Chemotherapy		0.0583	
No	2	7	
Yes	18	13	
Initial metastasis		0.7233	
Absent	15	14	
Present	5	6	

### Effect of circRNA-CIRH1A Silencing in Cell Proliferation and Apoptosis

**Fig. 2:**
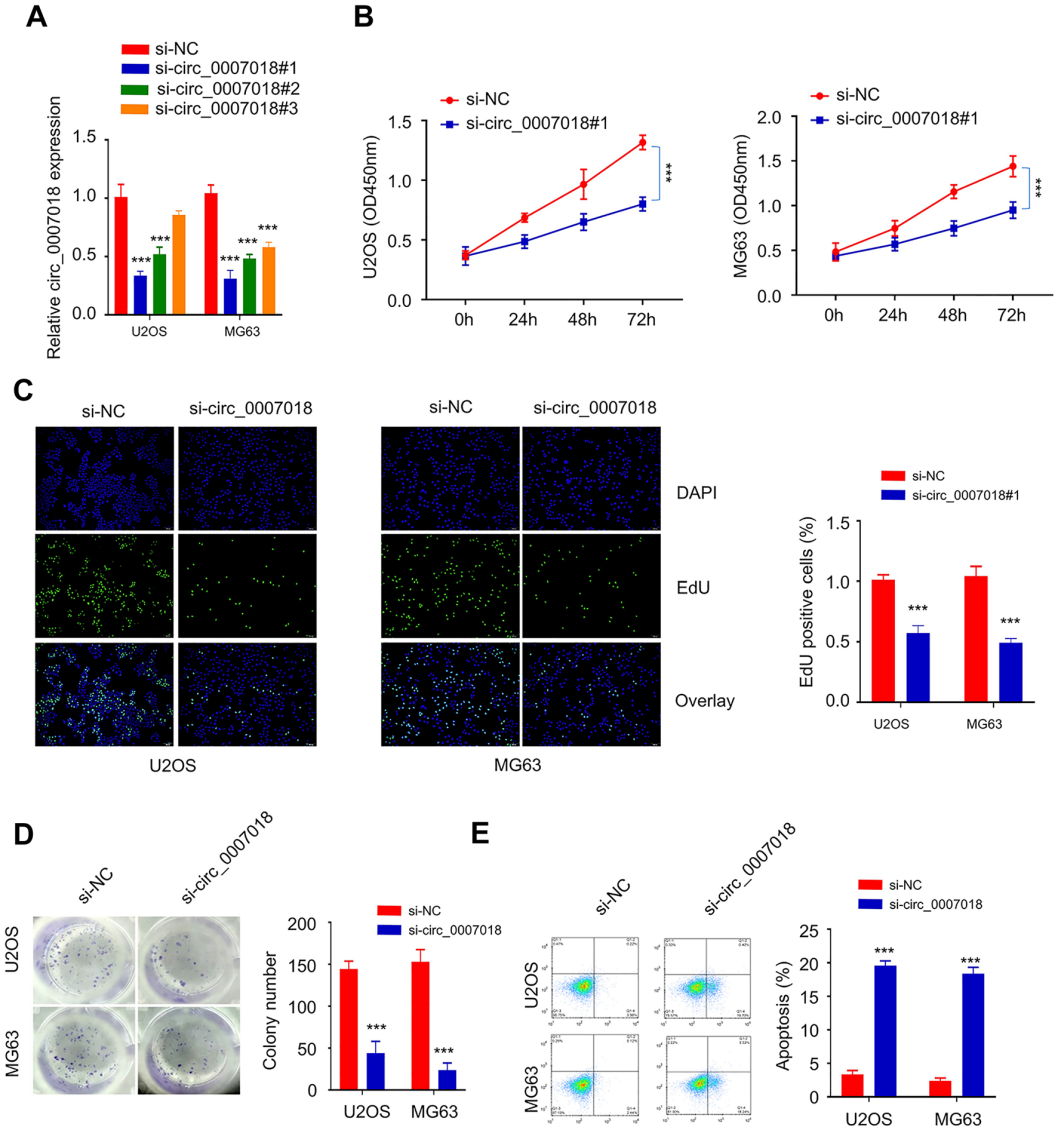
The effect of circRNA-CIRH1A silencing on OS cell phenotype. **A** CircRNA-CIRH1A was knocked down by three si-RNAs and the knockdown efficiency was assessed by qRT-PCR. **B** CCK8 proliferation assay in U2OS and MG-63 cells transfected with circRNA-CIRH1A siRNA. **C** EDU incorporation assay in cells upon circRNA-CIRH1A knockdown. **D** Knockdown of circRNA-CIRH1A reduced the colony formation ability of U2OS and MG-63 cells. **E** The apoptotic events in U2OS and MG-63 cells upon circRNA-CIRH1A silencing were determined by flow cytometry. *p < 0.05, **p < 0.01, ***p < 0.001

Two OS cell lines (U2Os and MG-63) with high circRNA-CIRH1A expression were selected for knockdown experiment. Compared to the si-NC group, the transfection of si- circRNA-CIRH1A#1, #2 or #3 each significantly downregulated the expression level of circRNA-CIRH1A*,* and si-circRNA-CIRH1A#1 with the strongest silencing effect was selected for further experiment (Fig. 2A, *p* < 0.001). After si-circRNA-CIRH1A transfection, CCK-8 proliferation assay was performed to assess the cellular proliferation after circRNA-CIRH1A knockdown. The results showed that circRNA-CIRH1A knockdown suppressed cell proliferation in U2Os and MG-63 cells (Fig. 2B, *p* < 0.001). Furthermore, the EDU incorporation assay demonstrated that circRNA-CIRH1A knockdown impaired the DNA synthesis in U2Os and MG-63 cells (Fig. 2C, *p* < 0.001). The impaired cell proliferation was further manifested by the inhibition of colony formation after circRNA-CIRH1A knockdown (Fig. 2D, *p* < 0.001). In contrast, circRNA-CIRH1A knockdown significantly promoted apoptosis in U2Os and MG-63 cells (Fig. 2E, *p* < 0.001). These data indicate that circRNA-CIRH1A upregulation support cell proliferation and survival in OS cancer.

To further validate the role of circRNA-CIRH1A, we also applied shRNAs ro stably silence circRNA-CIRH1A (Supplementary Fig. 1A), and circRNA-CIRH1A #1 with stronger knockdown efficiency was used for functional experiments. In U2Os and MG-63 cells, stable circRNA-CIRH1A knockdown also impaired cell proliferation, as evidenced by CCK-8 proliferation assay, EdU incorporation assay and colony formation assay (Supplementary Fig. 1B-1D). In the meanwhile, there was an increase in apoptotic events upon stable circRNA-CIRH1A knockdown (Supplementary Fig. 1E).

### Effect of circRNA-cirH1a Silencing in Tumorigenesis

**Fig. 3:**
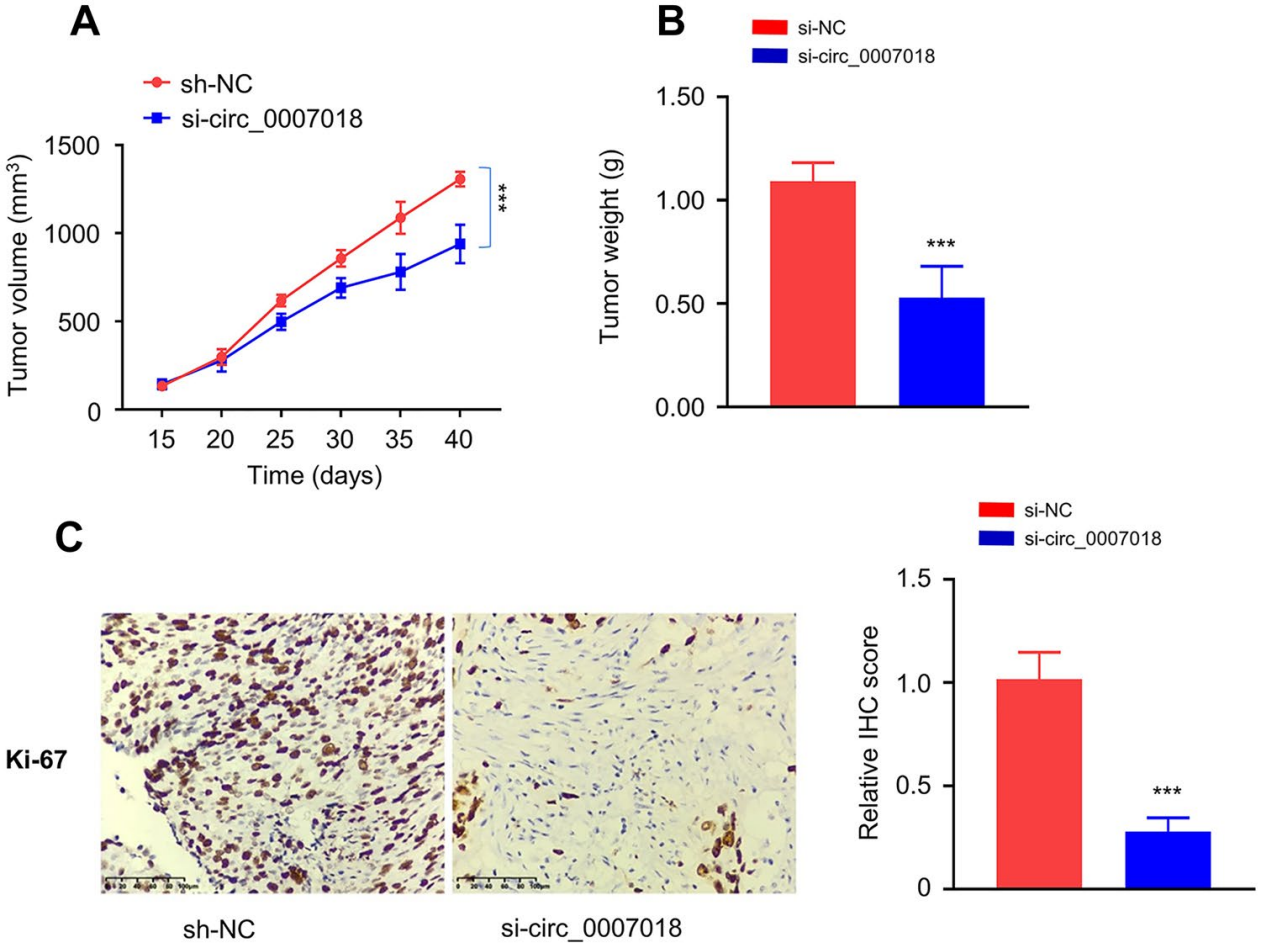
Silencing circRNA-CIRH1A impairs tumorigenesis of U2OS cells in nude mice. **A** The volume of subcutaneous tumorigenesis of U2OS cells with si-circRNA-CIRH1A silencing or control si-NC in nude mice. **B** The tumor weight was compared between si-circRNA-CIRH1A and si-NC group. **C** Immunohistochemical staining of Ki-67 in subcutaneous tumors sections in si-circRNA-CIRH1A and si-NC group. N = 6 in each group. *p < 0.05, **p < 0.01, ***p < 0.001

To examine the role of *circRNA-cirH1a in tumorigenesis,* U2OS cell transfected with si-circRNA-CIRH1A or si-NC were injected into immunodeficient nude mice. We monitored the tumor growth by measuring the tumor volume as well as the tumor weight at the end of the experiment. Knocking down circRNA-CIRH1A significantly reduce subcutaneous tumor volume and mass (Fig. 3A and 3B, *p* < 0.001). Further, the immunochemical staining of Ki67 in the tumor sections revealed that circRNA-CIRH1A silencing reduced the cells stained with Ki-67 (Fig. 3C, *p* < 0.001), which suggest that a high level of circRNA-CIRH1A expression is required for the sustained proliferation of U2OS cells in vivo.

### Effect of circRNA-CIRH1A Silencing on Cell Migration and Invasion

**Fig. 4:**
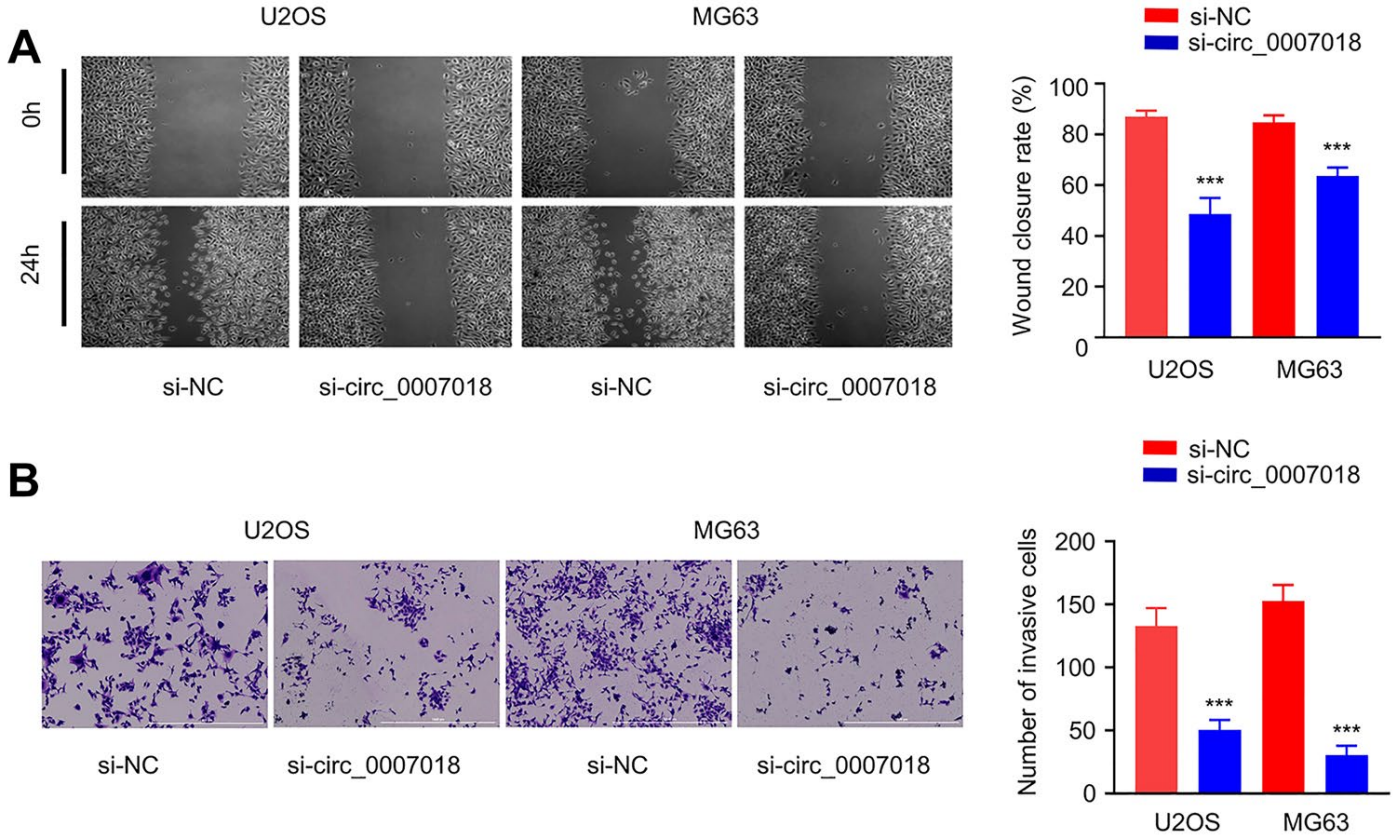
Silencing circRNA-CIRH1A impairs cell migration and invasion. **A** Would-healing assay to detect the migratory ability in U2OS and MG-63 cells upon circRNA-CIRH1A silencing. **B** Transwell invasion assay in U2OS and MG-63 cells upon circRNA-CIRH1A silencing. *p < 0.05, **p < 0.01, ***p < 0.001

We further investigated the role of circRNA-CIRH1A in cell migration and proliferation. The would-healing assay showed that circRNA-CIRH1A silencing impaired cell migration (Fig. 4A, *p* < 0.001), and the transwell invasion assay demonstrated an impaired cell invasion ability after circRNA-CIRH1A silencing (Fig. 4B, *p* < 0.001). Therefore, a high level of circRNA-CIRH1A expression also supports the migratory phenotype of OS cells.

### CircRNA-CIRH1A Silencing Impairs the Activation of PI3K/AKT and JAK2/STAT3 Signaling Pathways

**Fig. 5:**
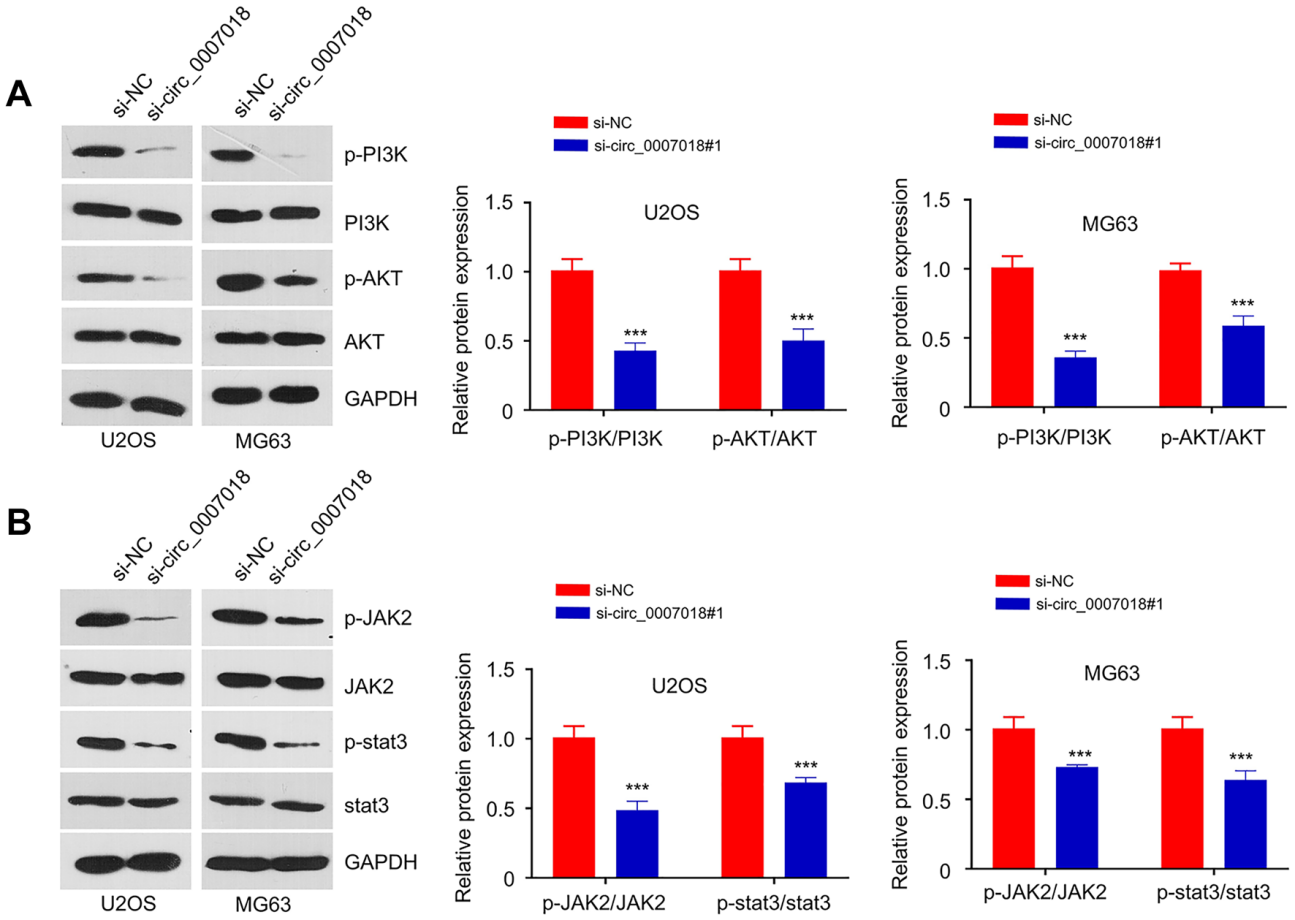
Silencing circRNA-CIRH1A impairs the activation of PI3K/AKT and JAK2/STAT3 pathways. **A** The relative protein levels of p-PI3K, PI3K, p-Akt, and Akt were measured by Western blot in U2OS and MG-63 cells upon circRNA-CIRH1A silencing. **B** The protein ratio of p-JAK2/JAK2 and p-STAT3 / STAT3 were detected by Western blotting after the knockdown of circRNA-CIRH1A. *p < 0.05, **p < 0.01, ***p < 0.001

As PI3K/AKT and JAK2-STAT3 signaling is involved in cell proliferation and survival, we then examined the activation status of these signaling pathways after CircRNA-CIRH1A *silencing.* The protein levels of p-PI3K, PI3K, p-AKT, AKT, p-JAK2, JAK2, p-STAT3, and STAT3 were compared in the si-NC group and si-circRNA-CIRH1A group of U2OS and MG-63 cells. The results demonstrated that the levels of p-PI3K, p-AKT, p-JAK2, p-STAT3 were significantly lower in the si-circRNA-CIRH1A group (Fig. 5, *p* < 0.001), indicating that silencing circRNA-CIRH1A impairs the activation of PI3K/AKT and JAK2/STAT3 signaling pathways.

### CircRNA-CIRH1A Competitively Binds to miR-1276 in OS Cells

**Fig. 6:**
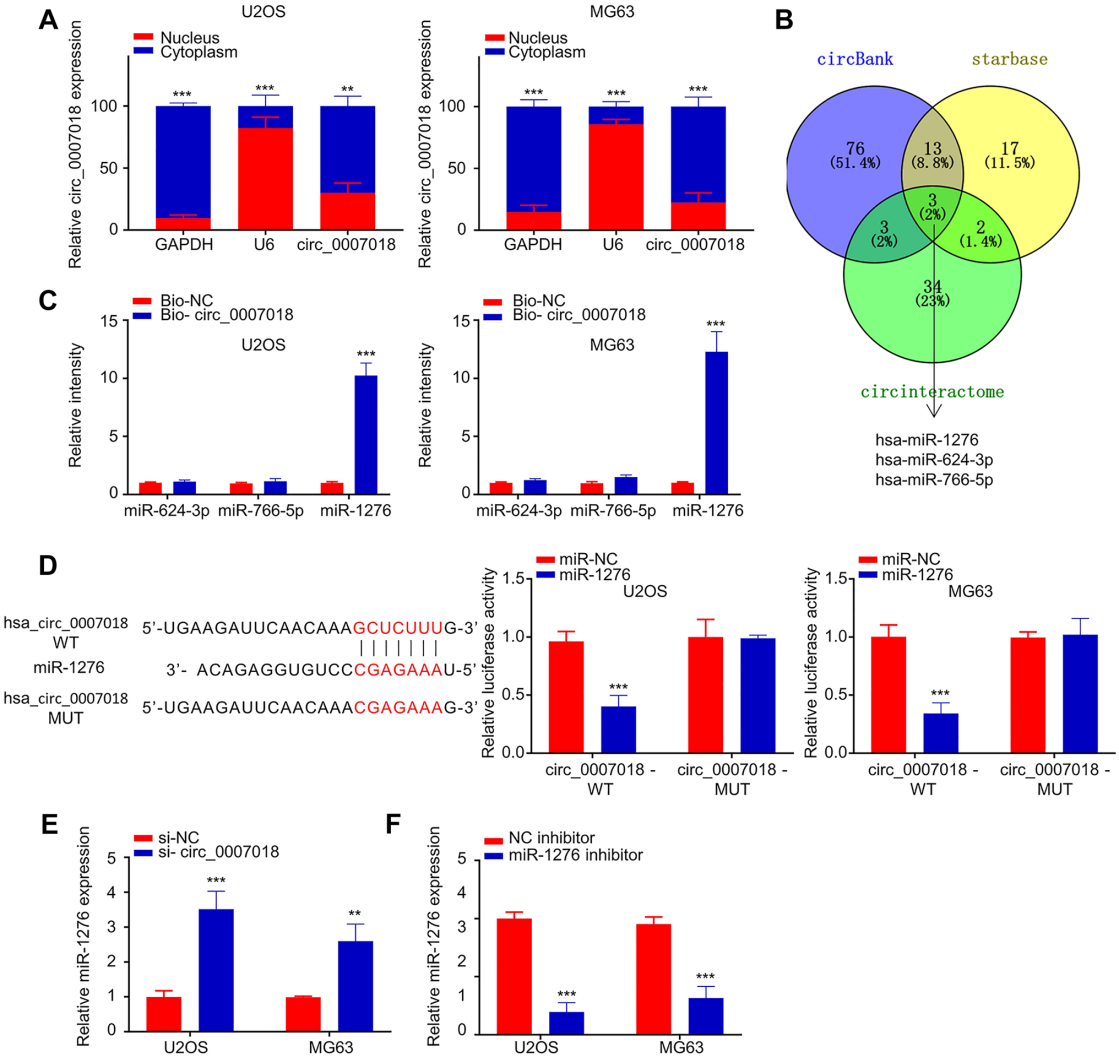
CircRNA-CIRH1A sponges miR-1276. **A** The relative level of circRNA-CIRH1A was analyzed in the cytoplasm and nuclear fraction of U2OS and MG63 cells. GAPDH and U6 were used as the marker for the cytoplasm and nuclear fraction. **B** The binding targets of circRNA-CIRH1A was predicted by three databases (circBank, Starbase and circinteractome) (**C**) RNA pull-down assay using biotin-labeled circRNA-CIRH1A revealed the interaction between circRNA-CIRH1A and miR-1276. **D** The interaction between circRNA-CIRH1A and miR-1276 was validated by dual-luciferase reporter assay using the luciferase reporter containing WT or mutated (MUT) binding site. **E** miR-1276 level was detected in U2OS and MG63 cells upon circRNA-CIRH1A silencing. **F** miR-1276 level was detected in U2OS and MG63 cells upon the transfection of iR-1276 inhibitor. *p < 0.05, **p < 0.01, ***p < 0.001

To identify the downstream target of circRNA-CIRH1A, we first analyzed the location of circRNA-CIRH1A. CircRNA-CIRH1A was predominantly localized in the cytoplasm of U2OS and MG63 cells (Fig. 6A, *p* < 0.001 or *p* < 0.01). We then searched the databases (circBank, Starbase and circinteractome), and found that three miRNAs are potential targets for circRNA-CIRH1A (Fig. 6B). Next, we performed RNA pull-down analysis using biotin-labeled circRNA-CIRH1A. The results demonstrated that circRNA-CIRH1A could significantly enrich miR-1276 (Fig. 6C, *p* < 0.001). To further validate the interaction between circRNA-CIRH1A and miR-1276, we performed dual-luciferase reporter assay using reporter containing wild type (WT) binding site or mutated binding site (MUT). The results showed that the presence of miR-1276 mimic significantly inhibited the luciferase activity, while no effect was observed for the MUT reporter (Fig. 6D, *p* < 0.001). In the meanwhile, we also observed that circRNA-CIRH1A silencing significantly increased the level of miR-1276 in U2OS and MG63 cells, while the transfection of miR-1276 inhibitor reduced miR-1276 level (Fig. 6E and 6F, *p* < 0.001). Together, these results indicate that circRNA-CIRH1A competitively binds to miR-1276 in OS cells.

### miR-1276 Mediates the Effect of circRNA-CIRH1A on PI3K/AKT and JAK2/STAT3 Signaling Pathways

**Fig. 7:**
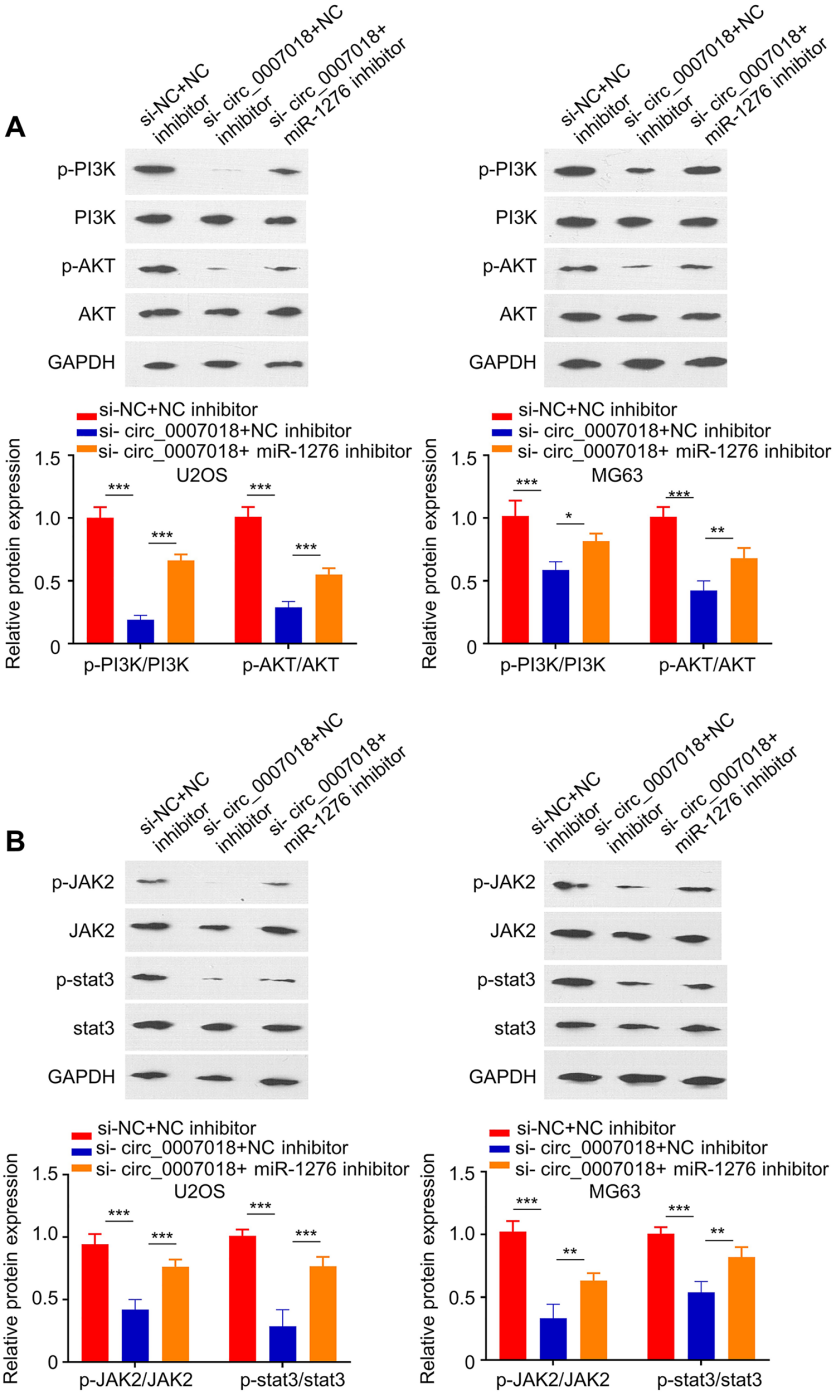
CircRNA-CIRH1A regulates PI3K/AKT and JAK2/STAT3 pathway by targeting miR-1276. U2OS and MG63 cells were transfected with circRNA-CIRH1A, or circRNA-CIRH1A and miR-1276 inhibitor. **A** The relative protein levels of p-PI3K, PI3K, p-Akt, and Akt were measured by Western blot. **B** The protein levels of p-JAK, JAK2, p-STAT3, and STAT3 were detected by Western blotting. *p < 0.05, **p < 0.01, ***p < 0.001

Next, we sought to determine whether miR-1276 mediates the effect of circRNA-CIRH1A on PI3K/AKT and JAK2/STAT3 signaling pathways. U2OS and MG63 cells were transfected with circRNA-CIRH1A siRNA as well as miR-1276 inhibitor. Since we observed that circRNA-CIRH1A silencing significantly increased the level of miR-1276 in U2OS and MG63 cells, while the transfection of miR-1276 inhibitor reduced miR-1276 level (Fig. 6E and 6F), the transfection of miR-1276 inhibitor was set to prevent the upregulation of miR-1276 upon circRNA-CIRH1A knockdown. We observed that circRNA-CIRH1A silencing impaired the activation of PI3K/AKT and JAK2/STAT3 signaling pathways, while the presence of miR-1276 inhibitor partially rescued the inhibitory effect (Fig. 7A, U2OS *p* < 0.001; MG63 *p* < 0.001, *p* < 0.01 or *p* < 0.05). Similar results were observed for JAK2/STAT3 signaling pathway (Fig. 7B, U2OS *p* < 0.001; MG63 *p* < 0.001 or *p* < 0.01). Together, these data imply that circRNA-CIRH1A regulates the activity of PI3K/AKT and JAK2/STAT3 signaling pathways through targeting miR-1276.

## Discussion

Osteosarcoma is the most common malignancy of bone which originates from mesenchymal tissue [23]. Early diagnosis is the critical to a better outcome of OS therapy [24]. However, the early symptoms of OS are not apparent, rendering it difficult in the early diagnosis of OS patients [2, 3]. In general, radical osteotomy is the mainstay of OS treatment. The exploration of the molecular mechanisms underlying the progression of OS could provide insights into the development of novel treatment strategies [25, 26]. Recent studies have implicated circRNAs in the pathological progression of OS. For example, the deregulation of circRNAs can contribute to tumor progression by regulating proliferation, invasion, migration, and apoptosis of OS cells, which are also correlated with the survival rate of OS patients [27–29].

In this study, we reported the upregulation of circRNA-CIRH1A in both OS tissues and OS cell lines. Moreover, the overall survival of OS patients with high circRNA-CIRH1A expression was shorter than the low-expression group, indicating that circRNA-CIRH1A was closely related to the progression of osteosarcoma. However, the mechanisms remain unclear [28, 29]. To further clarify the molecular mechanism underlying the oncogenic roles of circRNA-CIRH1A, we performed loss-of-function study by silencing circRNA-CIRH1A. We observed that silencing circRNA-CIRH1A impaired cell proliferation, migration, and invasion ability, while promoted the apoptosis events. These results suggest that circRNA-CIRH1A upregulation contributes to the malignant phenotype of OS cells. The oncogenic role of circRNA-CIRH1A was further supported by the in vivo tumorigenesis assay in nude mice. These results add novel evidence of circRNA-CIRH1A as an oncogenic circRNA in OS progression [30, 31].

Phosphatidylinositol 3-kinase (PI3K) signaling plays a vital role in the proliferation, differentiation, and apoptosis of tumor cells [16–18]. In recent years, the aberrant activation of PI3K/Akt pathway is implicated in the occurrence and development of tumors [32]. Akt activation triggers nitric oxide synthase, which produces nitric oxide to stimulate vascular dilation and formation, and promotes the proliferative survival and malignant progression of tumor cells [33]. The activation of JAK2/STAT3 signaling pathway also mediates the malignant progression in multiple cancers [19–22]. Therefore, the mechanisms underlying the activation of PI3K-Akt and JAK2/STAT3 signaling pathways in tumors become a research hotspot [34–36]. Our data suggests that circRNA-CIRH1A could regulate the activation of PI3K-Akt and JAK2/STAT3 signaling pathways. CircRNA-CIRH1A competitively binds to miR-1276 in OS cells, and the downregulation of miR-1276 activity contributes to the activation of PI3K-Akt and JAK2/STAT3 signaling pathways. However, it remains to be studied how miR-1276 suppresses the activation of PI3K-Akt and JAK2/STAT3 signaling pathways, and whether PI3K-Akt and JAK2/STAT3 signaling pathways are the major factors contributing to the oncogenic roles of CircRNA-CIRH1A in OS cells.

## Conclusion

In summary, we reported that circRNA-CIRH1A upregulation is implicated in the malignant phenotype of OS cells. A high level of circRNA-CIRH1A expression sustains the proliferation, invasion, and migration of OS cells in vitro, and promotes tumorigenesis in vivo. Interestingly, we showed that circRNA-CIRH1A regulates PI3K/AKT and JAK2/STAT3 signaling pathways by sponging miR-1276. Future studies are required to elucidate how miR-1276 suppresses the activation of PI3K-Akt and JAK2/STAT3 signaling pathways.

## Supplementary Information

Below is the link to the electronic supplementary material.

Supplementary Fig 1. (A) CircRNA-CIRH1A was transduced with lentivirus carrying shRNAs targeting CircRNA-CIRH1A. The silencing efficiency was assessed by qRT-PCR. (B) CCK8 proliferation assay in U2OS and MG-63 cells upon stable circRNA-CIRH1A knockdown. (C) EDU incorporation assay in cells upon stable circRNA-CIRH1A knockdown. (D) Stable knockdown of circRNA-CIRH1A reduced the colony formation ability in U2OS and MG-63 cells. (E) The apoptotic events in U2OS and MG-63 cells upon stable circRNA-CIRH1A knockdown were determined by flow cytometry. *P<0.05, **P<0.01, ***P<0.001. (TIF 1324 kb)

## Data Availability

All related data are available upon reasonable request to corresponding author via email.
