# Supplementary figures and images for: CircRNA-CIRH1A Promotes the Development of Osteosarcoma by Regulating PI3K/AKT and JAK2/STAT3 Signaling Pathways
Source: Mol Biotechnol. 2023 Aug 23;66(9):2241–53. doi: 10.1007/s12033-023-00812-0 (PMC11424664; doi:10.1007/s12033-023-00812-0)

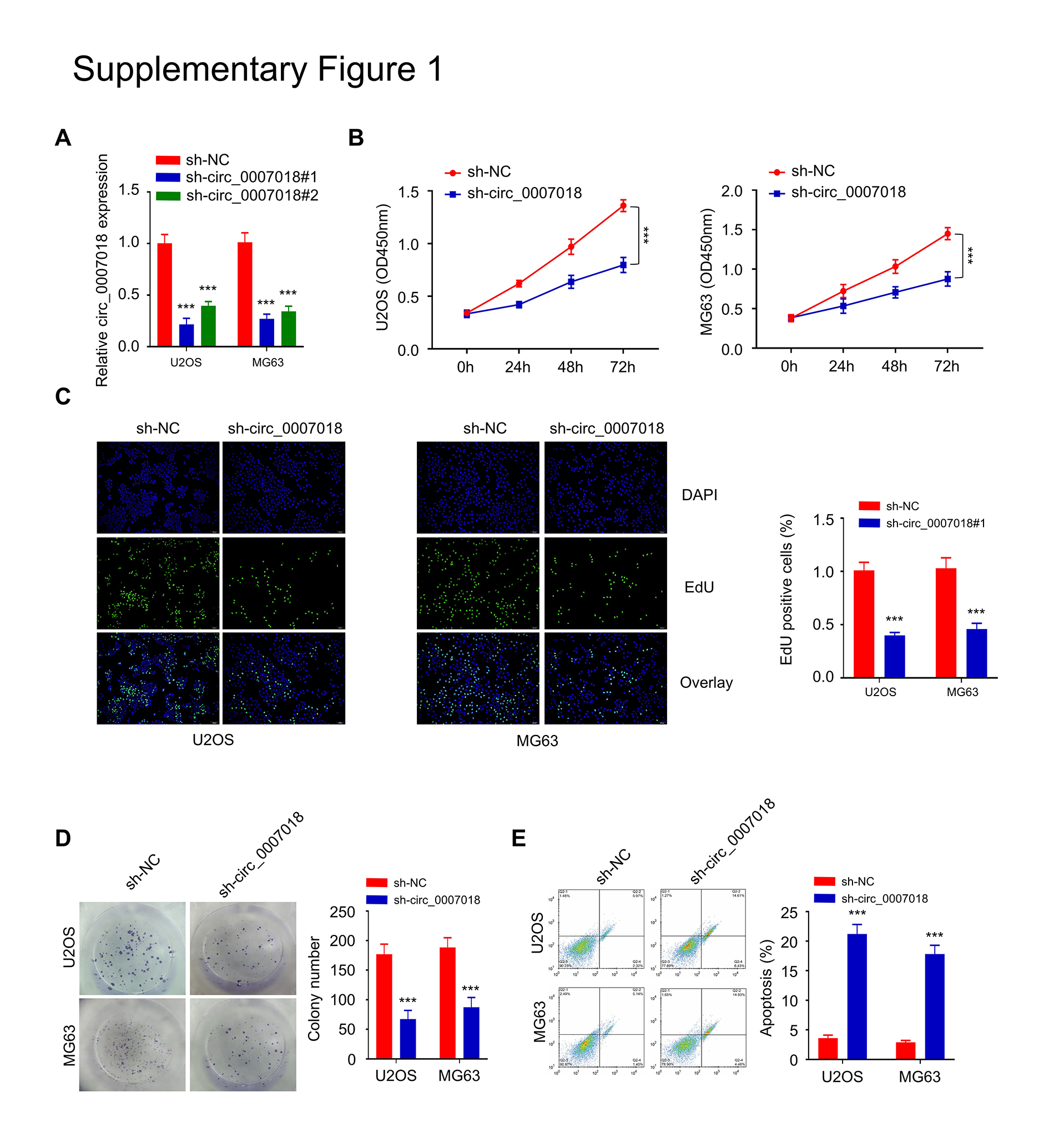

Supplement: Supplementary file 1 — Supplementary Fig 1. (A) CircRNA-CIRH1A was transduced with lentivirus carrying shRNAs targeting CircRNA-CIRH1A. The silencing efficiency was assessed by qRT-PCR. (B) CCK8 proliferation assay in U2OS and MG-63 cells upon stable circRNA-CIRH1A knockdown. (C) EDU incorporation assay in cells upon stable circRNA-CIRH1A knockdown. (D) Stable knockdown of circRNA-CIRH1A reduced the colony formation ability in U2OS and MG-63 cells. (E) The apoptotic events in U2OS and MG-63 cells upon stable circRNA-CIRH1A knockdown were determined by flow cytometry. *P<0.05, **P<0.01, ***P<0.001. (TIF 1324 kb) [file 12033_2023_812_MOESM1_ESM.tif]
